# Reframing sepsis research through translational integrative models

**DOI:** 10.1515/jtim-2026-0017

**Published:** 2026-02-18

**Authors:** Dandan Zhu, Krzysztof Laudanski

**Affiliations:** Department of Anesthesiology and Perioperative Care, Mayo Clinic, Rochester, MN, USA; Department of Critical Care Medicine, the Second Hospital of Dalian Medical University, Dalian, Liaoning Province, China

## Introduction

The uniqueness and complexity of human sepsis advocates for novel *in vitro*, *ex vivo*, and computational models. Animal models were considered foundational, but they have been facing scrutiny for their limited and inconsistent translational value.^[1]^ Concomitantly, methodological approaches integrating multiple molecular layers and multi-organ complexity augment these alternatives to current animal models.^[2]^ Our review focuses on clinically relevant translational models for sepsis research.

## Limitations of current animal models in sepsis research

Mice and rats are the most commonly used animal models of sepsis.^[1,3]^ Pigs, sheep, and non-human primates are sometimes chosen because of their closer similarities to human cardiovascular and neuronal functions.^[1,4,5]^ However, the human immune system’s unique features and interindividual heterogeneity make it difficult to apply findings from these models consistently.^[1]^ Moreover, animal models designed to mimic sepsis- such as cecal ligation and puncture (CLP) — often fail to accurately reflect clinical realities, representing only a narrow subset of causes, such as peritoneal sepsis resulting from perforation, as seen in CLP. Animal models are rarely used to study the chronic consequences of sepsis due to the high costs and effort required.^[1,3]^ Additionally, ethical concerns persist, particularly given the limited predictive accuracy of these models for human outcomes.^[6]^ Although animal models remain useful for preliminary investigations, emerging policies and scientific trends strongly advocate for human-relevant alternatives.^[1,7]^

## Advances in human-relevant ex vivo models

Primary cell cultures offer the advantage of preserving donor-specific physiological characteristics and intercellular interactions, while enabling high-content analyses of cellular responses to pathogens or drugs at both the cellular and molecular levels.^[8]^ They are relatively inexpensive. Their simplicity limits their use to early-stage and preliminary evaluations focused on dose response and toxicity across different cell lines. However, the readouts of these experiments reflect primary cell characteristics and the effects of mutations associated with immortalization. On the downside, they lack spatial architecture and any significant complexity. In addition, the artificial conditions required for culture introduce variables that may influence clinical translation. Therefore, this model is suitable primarily for preliminary pharmacodynamic and pharmacokinetic assessments. Organoids are three-dimensional, self-organizing structures that recapitulate tissue-specific architecture, allowing them to model spatial interactions.^[9]^ So, they enable the study of cell–cell and cell–matrix interactions of sepsis pathophysiology. Scalability from a few cells to a complex system allows the discovery of synergistic features as the system complexity grows. However, they cannot fully replicate the dynamic fluid microenvironment, as their ability to model the interplay among multiple factors and organs is limited. A significant limitation of iPSC-derived organoids is the progressive loss of cellular heterogeneity over prolonged *in vitro* culture. Their role in the research pipeline is to deliver preliminary data and explore the interactive aspects of sepsis at very initial steps. Organ-on-chip devices integrate multiple human tissues to mimic better the complexity of human homeostasis than traditional static *ex vivo* models.^[10]^ They constrained by limited *ex vivo* viability of tissues and cellular systems, high operational burden, and poor scalability, resulting in unfavorable suitability for large-scale applications (*e.g*. drug screening). Their place in the research pipeline is similar to that of organoids, but scalability remains a substantial obstacle, placing organoids in a comparatively more practical position than organ-on-chip platforms for exploratory studies.

## Advanced human-relevant systems and humanized models

Whole blood assays, in a way, resemble organoids or organ-on-chips, where elements of blood can be observed in their complexity of immune and humoral components. This methodology has a relevant human illness context..^[11,12]^ They are cost-effective. However, because blood is obtained at a specific point, reflecting a particular stage of the medical condition, it constitutes a static, time-limited assay. Metabolic instability is another limitation. Whole-blood assays cannot capture organ-derived cross-talk mechanisms. Leukocyte heterogeneity will affect the variability of the results. Simulating the complex hydrodynamics of human blood flow in these systems requires a careful, technically demanding setup. In the research pipeline, they represent an early step in testing. Perfused organ systems recreate near-physiologic organ interaction, allowing real-time assessment of metabolic activity, drug metabolism, and immune responses.^[13]^ Though they may lack pulsatile flow, they potentially represent one of the more physiologically aligned *ex vivo* platforms. Their downside is technical complexity, limited availability, and risks such as hemolysis during prolonged perfusion. They are useful to study sepsis interactions in complex, multicellular, and histological systems. Humanized animal models are generated by engrafting immunodeficient animals with human immune cells or tissues.^[14,15]^ Technically, these models enable the investigation of human-specific immune regulation, host-pathogen interactions, and therapeutic interventions within the context of sepsis.^[16]^ However, they are xenotransplant with an incomplete human immune system reconstruction, graft-versus-host complications, and high cost.^[14]^ The need for cytokine supplementation highlights their deficiency in capturing autonomous immune regulation, as the mouse host is unable to provide a sufficiently supportive environment for the transplanted immune system. Cost is significant due to the need for long-term maintenance and stem cell engrafting. Their place in the sepsis research pipeline is difficult to describe. On one side, they offer the complexity of human immunology. However, these advantages are tempered by the substantial limitations noted above. Consequently, their application in sepsis research remains relatively limited, reflecting significant limitations. Deceased donor models (DDM) are situations where patients are brain dead, but the organs and whole body cannot be used for transplantation. Thus, the human body can be donated to science and used for medical research and experimentation. This recapitulates human physiology more closely than any other available model, but the scarcity of opportunities significantly limits the technique’s impact. Also, the approach’s relative novelty has raised several ethical concerns. For some time, the application of this model to sepsis will be limited, as the ability to replicate experiments will remain severely constrained. This is a significant constraint as the heterogeneous nature of sepsis requires several replicates. It is difficult to project that donors’ availability will increase significantly, which further restricts the model’s applicability. Finally, what kind of model of sepsis should be applied to the deceased donor model? One could argue that a new model should be developed to mimic the most common clinical trajectory of sepsis, but the heterogeneity of sepsis in humans is vast. Standardization of the DDM is virtually nonexistent. Neuro-hormonal networks are disrupted, biasing potential experiments. Ethical concerns and donor availability may pose significant obstacles, substantially limiting the translational potential of this methodology.

## Computational approaches

Digital Twin technology — virtual representations of individual patients — leverage real-time clinical, omics, and sensor data to predict disease progression and simulate personalized treatment strategies using *in silico* simulation.^[17]^ By coupling patient-specific data with *in silico* modeling and advanced analytics, researchers could conduct virtual clinical trials and support individualized therapeutic planning. This represents a potentially valuable advancement, as it enables the “simulation” of an individual patient’s response before executing clinical interventions. However, clinical transability remains to be proven. Like other large data projects, digital twins are subjected to data bias. Currently, there is no universally recognized way to collect and validate data used for building digital twins. Their regulatory framework is in its infancy. Additional challenges include substantial computational requirements, difficulties in integrating heterogeneous multi-omics inputs, and the lack of prospective clinical validation. These are the reasons their translational value remains underdeveloped, despite the promise they could provide a convenient platform for testing and simulation of sepsis.

## Integrative approaches to analyze sepsis as the complex dysregulation of the immune system

If the above models (Table 1) are coupled with integrative “multi-omics” approaches—encompassing genomics, epigenomics, transcriptomics, proteomics, metabolomics, and microbiomics—they may transform understanding of sepsis heterogeneity and enable a more comprehensive methodology for studying the condition.^[7,18]^ As sepsis is an illness of dysfunction, recapitulating the dysregulation across different levels of molecular biology and involved systems, reductionist approaches will eventually become insufficient.^[19]^ However, the practical implementation of such integrative omics approaches needs to be developed. These methods demand substantial computational resources, and databases used to create such complex models must be unbiased and methodologically sound, a challenge for all omics techniques. Longitudinal modelling of multilevel omics accounts for pre-existing conditions, but such data are very scarce. Almost certainly, artificial intelligence methods will play a critical role in developing this field.^[18]^ Finally, the body of research relied upon to deliver the interactome is often biased or missing. So, despite significant promise, omics analysis is rare and usually limited to two, sometimes three, reactomes.^[19, 20, 21]^ In fact, their contribution to sepsis research may be similar to that of humanized mice. Both approaches seem to have significant potential, yet the methodology nuances are not immediately apparent and significantly limit their potential. A practical application would involve integrating organoid-derived epithelial injury signatures with patient whole-blood immune profiles to identify endotypes prone to sepsis-associated organ dysfunction—providing a concrete path toward resolving sepsis heterogeneity.

**Table 1:** Summary of sepsis novel models: Advantages and limitations

Model Type	Advantages	Limitations
Traditional animal models	• Established protocols • Allow systemic study of sepsis pathophysiology • Useful for initial hypothesis testing	• Limited human translatability due to species differences • Rarely applicable for modeling chronic trajectories • High cost, short observation windows, and limited predictive accuracy
Human-relevant *ex vivo* models (primary cells, organoids, organ-on-chip)	• Preserve donor-specific physiology • Organoids maintain 3D architecture and spatial interactions • Organ-on-chip more closely mimics multi-organ microenvironment	• Primary cells lack spatial architecture and may exhibit altered immune behavior • Loss of heterogeneity over time (organoids) • Limited ability to model multi-organ interactions (organoids) • High operational burden (organ-on-chip) • Lack of dynamic fluid microenvironment (organoids)
Advanced human-relevant systems (whole blood assays, perfused organ systems)	• Whole blood preserves native immune complexity • Perfused organ systems allow near-physiologic interaction and real‑time metabolic assessment	• Static nature of whole blood • Metabolic instability • Hydrodynamic complexity • Technical difficulty and hemolysis risk in perfusion platforms
Humanized animal models	• Enable *in vivo* study of human immune responses • Useful for evaluating human-specific immunomodulatory mechanisms • Allow host-pathogen interaction studies	• Incomplete and non-autonomous immune reconstitution • Cytokine supplementation requirement • GVHD risk • High inter-donor variability and limited reproducibility • Mouse host provides insufficient support for human immune cells • High cost
Deceased donor models	• More physiologically faithful compared with *ex vivo* or animal models	• Risk of pre-existing infections • Extremely limited availability • Severely limited reproducibility due to donor scarcity • High biological variability with minimal ability to control confounders • Unclear standardized sepsis induction model • Ethical and logistical constraints
Computational approaches (digital twins)	• Predict disease progression • Enable virtual trials • Integrate multi-modal data • May outperform traditional scoring systems in early detection	• Translational validity unproven • Data bias • Lack of standardized data collection and validation • Immature regulatory framework • Substantial computational requirements • Challenges integrating heterogeneous multi-omics inputs • Lack of prospective clinical validation

GVHD: graft-versus-host complications.

## Conclusions

To enhance the effectiveness and ethical integrity of sepsis research, the scientific community must actively redirect funding, infrastructure, and policy incentives toward the development, validation, and regulatory approval of human-relevant approaches.^[2,7,18]^ These efforts will reduce reliance on current animal models, potentially expanding the range of clinically translatable findings. However, a concerted effort must be made to standardize and reproduce the described models. Lab-to-lab variation in the maintenance and execution of these techniques impairs data reproducibility and their translation into clinically relevant interventions. The overarching impression from the above review is that these new approaches hold promise for enriching our sepsis methodology, yet none will deliver a single transformative breakthrough. They should be treated as complementary and synergistic approaches to studying sepsis, offering a more translational approach to this uniquely human illness. A structured pathway for analytical validation, clinical validation, and regulatory alignment will be essential for advancing these models toward broader adoption. Their ultimate translational utility may lie in integration with existing animal models, but such progress will require time and coordinated effort.
